# Dietary Quality and Associated Factors among Women of Reproductive Age in Six Sub-Saharan African Countries

**DOI:** 10.3390/nu16081115

**Published:** 2024-04-10

**Authors:** Amynah Janmohamed, Melissa M. Baker, David Doledec, Fatou Ndiaye, Ahmenan Claude Liliane Konan, Amoakon Leonce, Koffi Landry Kouadio, Maguette Beye, Delphine Danboyi, Theresia J. Jumbe, Alex Ndjebayi, Caleb Ombati, Benjamin K. Njenga, Romance Dissieka

**Affiliations:** 1 Helen Keller International, Vitamin A Supplementation Africa Regional Office, Nairobi 00800, Kenya; 2 Helen Keller International, Abidjan BP 1334, Côte d’Ivoire; 3 Helen Keller International, Yoff-Dakar BP 29898, Senegal; 4 Helen Keller International, Abuja 900001, Nigeria; 5 Helen Keller International, Dar es Salaam P.O. Box 34424, Tanzania; 6 Helen Keller International, Niamey BP 11728, Niger; 7 Helen Keller International, Nairobi 00800, Kenya

**Keywords:** Africa, dietary diversity, food groups, nutrition, women

## Abstract

The burden of micronutrient malnutrition is high among women of reproductive age (WRA) in sub-Saharan Africa. We examined the dietary quality and associated factors for WRA in Cameroon, Côte d’Ivoire, Kenya, Nigeria, Senegal, and Tanzania. Data were collected from women aged 15–49 years using representative Diet Quality Questionnaire surveys. The Minimum Dietary Diversity for Women (MDD-W), All-5 (key food group) consumption, noncommunicable disease risk (NCD-Risk), and Global Dietary Recommendation (GDR) indicators were assessed. Participants included N = 16,584 women [Cameroon: N = 2073; Côte d’Ivoire: N = 242; Kenya: N = 864; Adamawa State (Nigeria): N = 1283; Benue State (Nigeria): N = 1047; Nasarawa State (Nigeria): N = 1151; Senegal: N = 7232; Tanzania: N = 2692]. The MDD-W ranged from 43.0% in Tanzania to 81.4% in Côte d’Ivoire and was higher in urban, compared to rural, areas in Cameroon, Kenya, Nasarawa, Senegal, and Tanzania (*p* < 0.001). Increased education and wealth were positively associated with MDD-W in Kenya, Benue, Senegal, and Tanzania. Fewer than half of all women attained All-5 consumption. NCD-Risk scores ranged from 1.13 (95% CI: 1.08, 1.17) in Tanzania to 2.28 (95% CI: 2.16, 2.40) in Nasarawa, and women’s GDR scores ranged from 10.47 (95% CI: 10.40, 10.54) in Cameroon to 11.45 (95% CI: 11.25, 11.64) in Côte d’Ivoire. Our findings highlight key aspects of women’s diets in sub-Saharan African settings to enable greater awareness and more targeted responses to the specific areas needing the most improvement.

## 1. Introduction

The global burden of micronutrient malnutrition is high among women of reproductive age (WRA) [1,2]. Nutrient-poor diets, coupled with higher physiological needs, increase women’s vulnerability to adverse health and reproductive outcomes. In many resource-limited settings, a lack of adequately diversified diets and reliance on plant-based food sources are major contributors to women’s undernutrition [3,4]. Micronutrient deficiencies are of particular public health concern for women and children in sub-Saharan Africa (SSA). An analysis of 1990–2019 Global Burden of Disease data for 204 countries revealed that, despite a global decline in micronutrient deficiencies, rates remain high for WRA in SSA [5]. In 2019, South Asia and western Africa had the highest age-standardized prevalence of dietary iron deficiency, and the highest age-standardized disability-adjusted life year (DALY) rates for iodine deficiency and vitamin A deficiency were in Central Africa [5]. In addition, global estimates indicate one in three WRA are anemic, with anemia rates exceeding 40% among women in several SSA countries [6,7,8]. Moreover, available evidence suggests no country in SSA is positioned to achieve the global 50% anemia reduction target for WRA by 2025 [9]. Iron deficiency anemia, resulting from nutritional deficiencies due to inadequate diets, is considered a key contributor to the high prevalence of anemia among WRA in SSA [10].

Given the myriad challenges involved in implementing large-scale micronutrient surveys in low-resource settings, dietary intake assessments have been validated as a proxy measure of the probability of micronutrient adequacy [11,12]. However, current data on the quality of women’s diets tend to be limited for much of SSA. Therefore, the objective of this study was to examine the dietary quality and associated factors for WRA in Cameroon, Côte d’Ivoire, Kenya, Nigeria, Senegal, and Tanzania to add to existing knowledge on food consumption and potential diet-related contributors to women’s suboptimal nutrition in these settings.

## 2. Materials and Methods

During 2022, data were collected from women aged 15–49 years in each country using validated country-specific Diet Quality Questionnaire (DQQ) surveys. The DQQ is a standardized, publicly available, ready-to-use tool for collecting food-based data to assess population-level indicators of dietary adequacy, as well as indicators of protection against noncommunicable diseases (NCDs) [13,14]. Data were collected on prior 24 h consumption of sentinel foods from 29 standard food groups, with commonly consumed sentinel foods included in country-adapted questionnaires.

Except for Côte d’Ivoire, where simple random sampling was used, the surveys utilized a two-stage random cluster sampling design with enumeration areas stratified according to rural and urban location and selected using probability proportional to the size sampling. Households were randomly selected from lists created for each cluster. Research ethics approval was obtained from the respective institutions in each country, and informed consent was acquired from all survey participants. Enumerators were trained on the list-based DQQ methodology by Helen Keller Intl and administered country-specific DQQs to women in local languages. Data were collected for whether women did or did not consume items from each of the 29 food groups, and a binary code (0, 1) was applied for each group. Data were recorded using security-enabled mobile tablets, and field-based supervisors provided oversight for all survey activities.

We assessed five indicators of dietary adequacy that have been validated for the DQQ tool [14]. The Minimum Dietary Diversity for Women (MDD-W) was defined as the proportion of women who consumed items from ≥5 of the following 10 food groups the previous day or night: grains, white roots, tubers, and plantains; pulses; nuts and seeds; dairy; meat, poultry, and fish; eggs; dark green leafy vegetables; other vitamin A-rich fruits and vegetables; other vegetables; and other fruits. An All-5 indicator was calculated to determine the proportion of women who consumed foods from each of five categories: starchy staples; pulses, nuts, and seeds; animal-source foods; vegetables; and fruits. Three food-based indicators were measured to assess women’s protection against NCDs. An NCD-Protect score was calculated to determine women’s adherence to global dietary recommendations for healthy foods protective against NCDs and was based on consumption of items from nine food groups: whole grains; pulses; nuts and seeds; vitamin A-rich orange vegetables; dark green leafy vegetables; other vegetables; vitamin A-rich fruits; citrus; and other fruits. An NCD-Risk score was calculated to determine adherence to global recommendations for foods to limit or avoid and was based on women’s consumption of foods and beverages from eight food groups: soft drinks; baked/grain-based sweets; other sweets; processed meat; unprocessed red meat; deep-fried foods; fast foods and instant noodles; and packaged ultra-processed salty snacks. A Global Dietary Recommendations (GDR) score was calculated based on women’s consumption from the nine health-promoting food groups (NCD-Protect) and the eight food groups recommended to limit or avoid (NCD-Risk). Sociodemographic characteristics for women and households were also collected.

Univariate and bivariate analyses were conducted, and multivariable logistic regression models were used to examine associations between MDD-W and rural or urban residence, women’s age and education, and household wealth. Model covariates were selected based on a significant (*p* < 0.05) bivariate relationship and/or association with MDD-W based on prior knowledge. Sample-weighted data accounted for the cluster design. The results are presented as descriptive statistics and unadjusted and adjusted odds ratios with 95% confidence intervals. A two-sided *p*-value < 0.05 was considered statistically significant, and *p*-values were adjusted for multiple comparisons using Tukey’s HSD method. Separate analyses were conducted for each country and the three states in Nigeria, and the study was not designed to detect inter-country/state outcome differences. All analyses were performed using SPSS Complex Samples 26.0 (IBM Corp: Armonk, NY, USA).

## 3. Results

### 3.1. Participant Characteristics

Dietary intake data were collected for a total of N = 16,584 women [Cameroon: N = 2073; Côte d’Ivoire: N = 242; Kenya: N = 864; Adamawa State (Nigeria): N = 1283; Benue State (Nigeria): N = 1047; Nasarawa State (Nigeria): N = 1151; Senegal: N = 7232; Tanzania: N = 2692] (Table 1). The percentage of women in rural, compared to urban, areas was higher in all countries except for Cameroon. In Côte d’Ivoire, the study population included women only from rural households. Most women reported not having any formal education in Côte d’Ivoire and Senegal, and >80% of women had a primary or higher education in Cameroon, Kenya, Benue, Nasarawa, and Tanzania. Except for Adamawa, approximately 40% of the women resided in households classified as having very poor or poor wealth status (Table 1).

### 3.2. Food Group Consumption

Starchy staples (grains, white roots, tubers, plantains) were the most commonly consumed foods in all countries (Table 2). Flesh food (meat, poultry, fish) consumption ranged from 40.5% in Kenya to >75% in Côte d’Ivoire, Nasarawa, and Senegal. More than half of the women consumed dark green leafy vegetables the previous day or night in Côte d’Ivoire, Kenya, Adamawa, Nasarawa, Senegal, and Tanzania. Vitamin A-rich fruit and vegetable consumption was <50% in all countries except for Senegal. The percentage of women who achieved a minimally diverse diet the previous day ranged from 43.0% in Tanzania to 81.4% in Côte d’Ivoire. In all countries, fewer than half of the women attained All-5 consumption (Table 2).

### 3.3. Food Group Consumption and Socioeconomic Factors

Starchy staple consumption was similar between women in rural and urban areas in all regions except Adamawa and Nasarawa (Table 3). Women in urban, as compared to rural, areas had higher flesh food consumption in Cameroon (75.8% vs. 66.2%; *p* < 0.001), Kenya (48.3% vs. 36.0%; *p* < 0.001), Benue (84.9% vs. 71.1%; *p* = 0.001), Nasarawa (86.3% vs. 68.9%; *p* < 0.001), Senegal (92.5% vs. 75.7%; *p* < 0.001), and Tanzania (70.4% vs. 66.1%; *p* = 0.016). Women in rural, as compared to urban, areas had higher consumption of dark green leafy vegetables in Cameroon, Benue, and Senegal, while consumption was higher for women in urban areas in Nasarawa and Tanzania. Vitamin A-rich fruit and vegetable intake was higher among women in urban, as compared to rural, areas in Kenya (34.4% vs. 17.2%; *p* < 0.001), Nasarawa (53.6% vs. 43.8%; *p* = 0.001), Senegal (81.3% vs. 57.5%; *p* < 0.001), and Tanzania (53.2% vs. 11.0%; *p* < 0.001). The percentage of women attaining MDD the previous day was higher in urban, as compared to rural, areas in Cameroon (50.0% vs. 40.1%; *p* < 0.001), Kenya (71.9% vs. 52.5%; *p* < 0.001), Nasarawa (81.4% vs. 63.6%; *p* < 0.001), Senegal (67.4% vs. 46.7%; *p* < 0.001), and Tanzania (61.3% vs. 27.9%; *p* < 0.001). All-5 consumption was also higher among women in urban, as compared to rural, areas in Kenya, Nasarawa, Senegal, and Tanzania (Table 3).

Apart from Côte d’Ivoire, women in the wealthiest households had higher flesh food consumption, as compared to those in the poorest households, in all countries (Table 3). Women’s egg consumption was more than three times higher in the richest, as compared to the poorest, households in Nasarawa, Senegal, and Tanzania. A higher percentage of women attained MDD in the wealthiest households, as compared to the poorest households, in Cameroon (51.1% vs. 40.3%; *p* < 0.001), Kenya (70.7% vs. 46.2%; *p* < 0.001), Nasarawa (83.6% vs. 56.5%; *p* < 0.001), Senegal (66.7% vs. 31.0%; *p* < 0.001), and Tanzania (68.6% vs. 23.2%; *p* < 0.001). All-5 consumption was significantly higher among women in the richest, compared to the poorest, households in Kenya, Adamawa, and Nasarawa and was more than three times higher for women in wealthy vs. poor households in Senegal and Tanzania (Table 3).

In the multivariable analysis, rural residence was associated with a lower likelihood of MDD-W in Kenya (OR: 0.65; 95% CI: 0.44, 0.95) and Tanzania (OR: 0.62; 95% CI: 0.48, 0.80) (Table 4). Women with a primary-level education were more likely to attain MDD in Kenya and Benue, and having a secondary or higher education was associated with a higher likelihood of MDD in Kenya, Benue, Senegal, and Tanzania. Women in the highest wealth households were more likely to have a minimally diverse diet the previous day in Kenya (OR: 1.83; 95% CI: 1.21, 2.75), Benue (OR: 1.40; 95% CI: 1.03, 1.90), Nasarawa (OR: 3.58; 95% CI: 2.33, 5.52), Senegal (OR: 3.80; 95% CI: 3.34, 4.32), and Tanzania (OR: 4.59; 95% CI: 3.38, 6.22) (Table 4).

### 3.4. Noncommunicable Disease-Associated Food Consumption

Of the eight NCD-associated food groups, baked/grain-based sweets and other sweets were the most commonly consumed in all regions except Benue (Table 5). Soft drink intake ranged from 3.2% of women in Kenya to 31.9% in Nasarawa. Processed meat and packaged ultra-processed salty snack consumption were <5% in all settings, except for a slightly higher intake of salty snacks in Nasarawa. NCD-Risk scores were low across all countries and ranged from 1.13 (95% CI: 1.08, 1.17) in Tanzania to 2.28 (95% CI: 2.16, 2.40) in Nasarawa, and women’s GDR scores were >10 in all regions (Table 6).

## 4. Discussion

In this study, we characterized key aspects of women’s dietary quality in eight regions of SSA using indicators that reflect adherence to global dietary recommendations for nutrient adequacy and protection against NCDs. Our findings suggest a lack of dietary diversity and low consumption of key food groups for optimal health. Starchy staples were predominantly consumed, with a lower intake of animal-source foods, plant-based proteins, and vitamin A-rich fruits and vegetables, which is of critical concern for women’s health and nutrition. These findings are consistent with the available data showing a high percentage of dietary energy is obtained from cereals, roots, and tubers in SSA countries [15,16]. The ≤60% of women having a minimally diverse diet the previous day in six regions, coupled with fewer than half of all women consuming the five recommended daily food groups, suggest women are unlikely to meet their daily nutritional requirements in these settings. The much lower consumption of vitamin A-rich fruits and vegetables, compared to other fruits and vegetables, in all countries except Senegal suggests suboptimal vitamin A intake among WRA in these areas. While the high (>70%) reported consumption of flesh foods in five regions is an encouraging finding, small quantities may have precluded nutrient adequacy.

We observed lower MDD-W prevalence compared to available estimates derived from previous DQQ assessments conducted for women in Cameroon (46% vs. 53%), Kenya (60% vs. 69%), and Senegal (51% vs. 75%) [14]. Conversely, compared to the available data, we observed a 7% and 12–22% higher MDD-W prevalence for Tanzania and the three states in Nigeria, respectively [14]. The All-5 prevalence estimates were similar to the available data for Cameroon (22% vs. 25%) and Tanzania (20% vs. 18%), lower for Kenya (29% vs. 37%) and Senegal (17% vs. 31%), and 10–20% higher for Nigeria [14]. These contrasting findings could be attributed to geographic and/or seasonal factors impacting food availability and accessibility, particularly in the countries where the survey was conducted in the lean season. The higher MDD prevalence and consumption of flesh foods among women in urban and wealthier households indicate socioeconomic advantages to achieving greater food diversity and increased access to higher-quality food sources in these populations. High food costs, particularly for animal-source foods, reduce the affordability of healthy diversified diets for women who are especially vulnerable to undernutrition. The available data indicate that 61% of the population in Cameroon, 81% in Kenya, 96% in Nigeria, 46% in Senegal, and 88% in Tanzania are not able to attain what is globally considered a healthy diet [17]. In our study, >75% reported consumption of flesh foods, nuts and seeds, dark green leafy vegetables, and other vegetables, and the >80% MDD-W prevalence in Côte d’Ivoire was higher than expected but not entirely unanticipated. The DQQ survey in Côte d’Ivoire included only female farmers participating in a project to strengthen agricultural and livestock productivity and improve food security and nutrition, particularly through increasing the cultivation and consumption of vitamin A-rich foods. The positive association between women’s educational status and dietary quality observed in our study has been shown in other SSA settings [18,19,20,21]. In a study examining adult dietary patterns across 185 countries, increased education was linked to higher diet quality scores [4]. The study also highlighted the improvement in dietary quality between 1990 and 2018 in all regions except South Asia and SSA [4].

Women’s NCD-Protect, NCD-Risk, and GDR scores in our study are similar to the available data from DQQ assessments conducted in Cameroon, Kenya, Nigeria, and Tanzania [14]. Though validated methods for characterizing NCD and GDR scores are not available, the low NCD-Risk scores observed in our study likely indicate NCD-limiting dietary patterns among women in these settings. However, the high intake of sweet items, ranging from ~30 to 80% across our study sites, is concerning, as it indicates women’s daily sugar intake may be disproportionately high in some areas. The consumption of foods high in sugar, salt, and fat has shown an upward trend in low- and middle-income countries in the context of rapid nutrition transitions away from traditional foods to more increasingly accessible energy-dense and nutrient-lacking foods [22,23]. Women’s low NCD-Protect scores and All-5 consumption in our study suggest under-consumption of nutrient-rich health-promoting foods and, therefore, a lower likelihood of adequate micronutrient intake.

The strengths of our study include the eight multi-country samples and standard survey methodology implemented in all areas using the novel low-burden DQQ tool developed to capture both protective and unhealthy aspects of dietary intake. The DQQ is now being used in various countries to collect data that align with key population-level indicators apart from MDD-W, which, hitherto, has been the only low-burden food-based dietary indicator for women and is insufficient for assessing overall diet quality [24]. However, several limitations of the study should be considered. The DQQ tool elicits data using a list-based approach, whereby women are asked whether they did or did not consume sentinel items from 29 food groups, as opposed to an open recall method. Therefore, women may have consumed foods not included in the list-based questionnaires that could have contributed to their total food intake. However, as country-adapted DQQs have been designed to include the most typically consumed foods in amounts >15 g in each country [24,25], it is unlikely that this influenced our results. In Côte d’Ivoire, the data were collected only from women in rural areas, which precluded examining the influence of locality on women’s dietary intake. In Senegal, the dairy assessment was limited to cheese intake and, therefore, was not representative of all dairy products women may have consumed the previous day. NCD-Risk was not assessed for women in Senegal due to the limited data available for the NCD-associated food groups.

We were also limited in our ability to infer women’s nutritional intake due to the lack of data on the quantities of consumed foods. Cost-driven consumption of small quantities of nutrient-dense foods, such as meat and eggs, likely prevents women in poorer households from attaining their micronutrient requirements. Although obtaining data on food quantity and frequency would have enabled a more comprehensive understanding of women’s dietary intake in our study areas, the logistical challenges involved in collecting such data and the variable data quality observed for food measurements are important considerations [26,27]. We explored the influence of rural or urban residence and age, education, and household wealth predictors on women’s dietary diversity. However, as diet-related behaviors are multi-dimensional, other factors that may have affected women’s food choices and consumption patterns such as household size, household food security, women’s decision-making roles, intra-household food allocation practices, and knowledge-related determinants should be considered. Lastly, the findings from these cross-sectional surveys do not imply causality, and we were not able to pool country/state data due to sampling variations across the study sites.

A good-quality diet is characterized by adequate consumption of diverse nutrient-rich foods and limited intake of unhealthy foods. Our findings highlight gaps in women meeting global food recommendations for healthy diets, which have broader implications for women’s health and nutrition in these settings. Bridging these gaps requires the strengthening and prioritizing of community-based strategies to improve women’s dietary quality as part of ongoing policies and practices to reduce diet-associated risks for nutritionally vulnerable women in SSA. This should include direct education to encourage the consumption of nutrient-dense foods from a variety of food groups, as well as peer-to-peer groups, which have been shown to improve health and nutrition knowledge and outcomes in low- and middle-income countries [28]. In addition, more intensive local capacity building for nutrition should be implemented. Future studies should explore other limitations to achieving adequately diversified diets including knowledge-related determinants, cultural practices and intra-household dynamics, proximity to markets and food sellers, and seasonal fluctuations to better understand their impact on women’s diets. Also, examining associations between DQQ-derived indicators and nutrition outcomes, and exploring the mechanisms underlying these, could be considered.

## 5. Conclusions

Periodic dietary intake surveys are important for filling existing data gaps, better understanding the linkages between dietary diversity and nutrition-related outcomes, and informing nutrition-relevant policies and programs to reduce vulnerabilities and dietary inequities. The use of a standardized tool such as the DQQ enables comparable data collection and monitoring of dietary patterns across regions. Our findings highlight key aspects of women’s diets in sub-Saharan African settings to enable greater awareness and more targeted responses to specific areas needing the most improvement.

## Figures and Tables

**Table 1:** Participant characteristics.

	Cameroon N = 2073	Côte d’Ivoire N = 242	Kenya N = 864	Adamawa N = 1283	Benue N = 1047	Nasarawa N = 1151	Senegal N = 7232	Tanzania N = 2692
	*n* (%)	*n* (%)	*n* (%)	*n* (%)	*n* (%)	*n* (%)	*n* (%)	*n* (%)
Area								
Rural	823 (39.7)	242 (100.0)	547 (63.3)	1173 (91.4)	899 (86.6)	714 (62.4)	5838 (80.7)	1471 (54.6)
Urban	1248 (60.3)	0 (0.0)	317 (36.7)	110 (8.6)	139 (13.4)	431 (37.6)	1394 (19.3)	1221 (45.4)
Women’s age								
15–19 years	78 (3.8)	7 (2.9)	17 (2.0)	43 (3.4)	21 (2.0)	17 (1.5)	397 (5.5)	64 (2.4)
20–29 years	962 (46.4)	58 (24.0)	377 (43.6)	715 (55.7)	575 (54.9)	663 (57.6)	3873 (53.6)	1365 (50.7)
30–39 years	872 (42.0)	79 (32.6)	382 (44.2)	447 (34.8)	404 (38.6)	416 (36.1)	2530 (35.0)	981 (36.4)
≥40 years	161 (7.8)	98 (40.5)	88 (10.2)	78 (6.1)	47 (4.5)	55 (4.8)	432 (6.0)	282 (10.5)
Women’s education								
None	161 (7.8)	230 (95.0)	25 (2.9)	434 (33.8)	162 (15.5)	158 (13.7)	5243 (72.5)	345 (12.8)
Primary	475 (22.9)	12 (5.0)	429 (49.7)	349 (27.2)	424 (40.5)	395 (34.3)	1166 (16.1)	1811 (67.3)
Secondary/higher	1437 (69.3)	0 (0.0)	410 (47.4)	500 (39.0)	461 (44.0)	598 (52.0)	823 (11.4)	536 (19.9)
Household wealth								
Very poor	431 (20.8)	39 (16.2)	151 (17.6)	44 (3.4)	111 (10.6)	94 (8.2)	1417 (19.6)	588 (21.8)
Poor	415 (20.0)	48 (19.9)	161 (18.8)	0 (0.0)	264 (25.2)	331 (28.8)	1478 (20.4)	600 (22.3)
Medium	409 (19.7)	54 (22.4)	180 (21.0)	667 (52.0)	208 (19.9)	244 (21.2)	1429 (19.8)	517 (19.2)
Rich	417 (20.1)	51 (21.2)	148 (17.3)	282 (22.0)	239 (22.8)	220 (19.1)	1456 (20.1)	511 (19.0)
Very rich	401 (19.3)	49 (20.3)	217 (25.3)	290 (22.6)	225 (21.5)	262 (22.8)	1452 (20.1)	476 (17.7)

**Table 2:** Food group consumption, Minimum Dietary Diversity, and All-5 consumption for women of reproductive age.

	Cameroon N = 2073	Côte d’Ivoire N = 242	Kenya N = 864	Adamawa N = 1283	Benue N = 1047	Nasarawa N = 1151	Senegal N = 7232	Tanzania N = 2692
	*n* (%)	*n* (%)	*n* (%)	*n* (%)	*n* (%)	*n* (%)	*n* (%)	*n* (%)
Starchy staples	1966 (94.8)	241 (99.6)	853 (98.7)	1194 (93.1)	999 (95.4)	1078 (93.7)	7201 (99.6)	2648 (98.4)
Pulses	437 (21.1)	63 (26.0)	355 (41.1)	802 (62.5)	548 (52.3)	729 (63.3)	2075 (28.7)	1347 (50.0)
Nuts and seeds	826 (39.8)	215 (88.8)	74 (8.6)	725 (56.5)	666 (63.6)	784 (68.1)	3302 (45.7)	343 (12.7)
Dairy products	525 (25.3)	102 (42.1)	620 (71.8)	568 (44.3)	204 (19.5)	564 (49.0)	77 (1.1)	381 (14.2)
Meat, poultry, fish	1493 (72.0)	189 (78.1)	350 (40.5)	793 (61.8)	761 (72.7)	867 (75.3)	5711 (79.0)	1832 (68.1)
Eggs	468 (22.6)	66 (27.3)	58 (6.7)	237 (18.5)	197 (18.8)	283 (24.6)	769 (10.6)	199 (7.4)
Dark green leafy veg	770 (37.1)	191 (78.9)	615 (71.2)	699 (54.5)	505 (48.2)	656 (57.0)	3996 (55.3)	1511 (56.1)
Other vit A fruit/veg	653 (31.5)	92 (38.0)	203 (23.5)	470 (36.6)	360 (34.4)	547 (47.5)	4491 (62.1)	812 (30.2)
Other vegetables	1320 (63.7)	210 (86.8)	696 (80.6)	661 (51.5)	578 (55.2)	691 (60.0)	4075 (56.3)	1770 (65.8)
Other fruits	905 (43.7)	130 (53.7)	478 (55.3)	632 (49.3)	624 (59.6)	623 (54.1)	996 (13.8)	1004 (37.3)
MDD-W ^†^								
Yes	954 (46.0)	197 (81.4)	515 (59.6)	773 (60.2)	628 (60.0)	808 (70.2)	3663 (50.6)	1158 (43.0)
No	1119 (54.0)	45 (18.6)	349 (40.4)	510 (39.8)	419 (40.0)	343 (29.8)	3569 (49.4)	1534 (57.0)
All-5 ^‡^								
Yes	465 (22.4)	112 (46.3)	248 (28.7)	459 (35.8)	423 (40.4)	519 (45.1)	1261 (17.4)	526 (19.5)
No	1608 (77.6)	130 (53.7)	616 (71.3)	824 (64.2)	624 (59.6)	632 (54.9)	5971 (82.6)	2166 (80.5)

MDD-W, Minimum Dietary Diversity for Women. ^†^ Based on prior 24 h consumption of ≥5 of the following groups: grains, white roots, tubers, and plantains; pulses; nuts and seeds; dairy products; meat, poultry, and fish; eggs; dark green leafy vegetables; other vitamin A-rich fruits and vegetables; other vegetables; other fruits. ^‡^ Based on prior 24 h consumption of grains, white roots, tubers, and plantains; pulses, nuts, and seeds; animal-source foods; vegetables; fruits.

**Table 3:** Consumption of key food groups, Minimum Dietary Diversity, and All-5 consumption for women of reproductive age by household area and wealth status.

	Starchy Staples	Dark Green Leafy Veg	Vitamin A Fruit/Veg	Flesh Foods	Eggs	Dairy	Pulses, Nuts, Seeds	MDD-W ^†^	All-5 ^‡^
	%	%	%	%	%	%	%	%	%
Cameroon ^1^									
Rural	95.9	40.1	33.2	66.2	15.7	17.0	53.6	40.1	21.0
Urban	94.2	35.2	30.4	75.8	27.2	30.8	52.9	50.0	23.4
Very poor/poor	94.7	40.5	32.0	64.5	17.4	16.9	53.8	40.3	21.0
Medium	95.8	33.0	33.0	74.1	22.0	27.4	54.0	47.7	24.0
Rich/very rich	94.5	35.7	30.2	78.7	28.2	33.0	52.0	51.1	23.1
Côte d’Ivoire ^2^									
Very poor/poor	100.0	77.0	36.8	74.7	20.7	34.5	90.8	81.6	43.7
Medium	100.0	74.1	33.3	77.8	25.9	37.0	92.6	75.9	42.6
Rich/very rich	99.0	83.0	41.0	81.0	33.0	52.0	91.0	84.0	50.0
Kenya ^3^									
Rural	98.5	69.1	17.2	36.0	5.1	72.2	42.8	52.5	23.9
Urban	99.1	74.8	34.4	48.3	9.5	71.0	47.9	71.9	36.9
Very poor/poor	98.4	68.3	16.7	29.5	5.1	71.2	43.9	46.2	23.7
Medium	100.0	73.9	21.1	41.7	9.4	67.2	46.1	60.6	25.6
Rich/very rich	98.6	72.6	30.7	49.9	6.8	75.1	44.9	70.7	34.5
Adamawa ^4^									
Rural	92.4	53.8	36.4	61.3	17.6	43.7	80.6	60.0	35.5
Urban	100.0	61.8	39.1	67.3	28.2	50.0	69.1	62.7	38.2
Very poor/poor	88.6	65.9	22.7	43.2	13.6	45.5	86.4	65.9	18.2
Medium	90.0	57.3	34.8	55.2	14.8	39.6	79.8	54.1	31.2
Rich/very rich	97.0	50.3	39.9	71.0	23.1	49.7	79.0	67.0	42.5
Benue ^5^									
Rural	95.8	50.7	34.8	71.1	19.2	19.2	77.5	61.0	41.6
Urban	92.8	33.1	30.2	84.9	16.5	20.1	69.1	53.2	33.8
Very poor/poor	92.8	50.4	33.1	63.2	15.2	10.4	77.6	57.1	41.1
Medium	95.2	55.8	31.7	70.2	15.4	15.4	74.5	57.2	35.6
Rich/very rich	97.6	43.1	36.6	81.5	23.3	28.7	76.1	63.6	42.0
Nasarawa ^6^									
Rural	92.3	52.7	43.8	68.9	15.1	40.6	84.0	63.6	37.8
Urban	95.8	63.8	53.6	86.3	39.9	63.3	84.7	81.4	57.8
Very poor/poor	90.6	53.2	38.1	63.1	7.8	36.0	79.8	56.5	33.2
Medium	92.6	49.6	42.2	70.1	21.7	34.4	86.1	67.6	39.8
Rich/very rich	96.9	64.1	58.5	88.8	40.9	67.8	87.3	83.6	58.3
Senegal ^7^									
Rural	99.6	57.7	57.5	75.7	7.9	0.8	61.2	46.7	14.9
Urban	99.6	44.8	81.3	92.5	22.1	2.4	60.0	67.4	28.2
Very poor/poor	99.3	63.5	34.9	61.1	4.9	0.2	63.5	31.0	7.8
Medium	99.7	56.0	71.4	86.4	9.6	1.3	60.3	57.9	18.8
Rich/very rich	99.7	46.7	84.7	93.1	16.8	1.8	58.7	66.7	26.4
Tanzania ^8^									
Rural	98.2	50.8	11.0	66.1	3.7	11.1	47.8	27.9	11.1
Urban	98.5	62.5	53.2	70.4	11.8	17.8	62.9	61.3	29.7
Very poor/poor	98.0	49.0	9.0	59.3	3.5	8.8	50.1	23.2	9.6
Medium	99.0	52.2	22.2	76.4	3.9	12.6	53.4	39.7	15.1
Rich/very rich	98.5	66.8	59.8	74.3	14.0	21.5	60.8	68.6	33.8

MDD-W, minimum dietary diversity for women. ^†^ Based on prior 24 h consumption of ≥5 of the following groups: grains, white roots, tubers, plantains; pulses; nuts and seeds; dairy products; meat, poultry, fish; eggs; dark green leafy vegetables; other vitamin A-rich fruits and vegetables; other vegetables; and other fruits. ^‡^ Based on prior 24 h consumption of grains, white roots, tubers, plantains; pulses, nuts, seeds; animal-source foods; vegetables; and fruits. ^1^ Cameroon rural vs. urban: dark green leafy vegetables *p* = 0.023; flesh foods *p* < 0.001; eggs *p* < 0.001; dairy *p* < 0.001; MDD *p* < 0.001. Cameroon wealth status: dark green leafy vegetables poor vs. medium *p* = 0.026; flesh foods poor vs. medium *p* = 0.001, poor vs. rich *p* < 0.001; eggs poor vs. rich *p* < 0.001, medium vs. rich *p* = 0.035; dairy poor vs. medium *p* < 0.001, poor vs. rich *p* < 0.001; MDD poor vs. medium *p* = 0.037, poor vs. rich *p* < 0.001. ^2^ Côte d’ Ivoire dairy poor vs. rich *p* = 0.041. ^3^ Kenya rural vs. urban: other vitamin A-rich fruits and vegetables *p* < 0.001; flesh foods *p* < 0.001; eggs *p* = 0.014; MDD *p* < 0.001; All-5 *p* < 0.001. Kenya wealth status: other vitamin A-rich fruits and vegetables poor vs. rich *p* < 0.001, medium vs. rich *p* = 0.034; flesh foods poor vs. medium *p* = 0.020, poor vs. rich *p* < 0.001; MDD poor vs. medium *p* = 0.004, poor vs. rich *p* < 0.001, All-5 poor vs. rich *p* = 0.005. ^4^ Adamawa rural vs. urban: starchy staples *p* = 0.003; eggs *p* = 0.006; pulses, nuts, seeds *p* = 0.004. Adamawa wealth status: starchy staples medium vs. rich *p* < 0.001; dark green leafy vegetables medium vs. rich *p* = 0.039; flesh foods poor vs. rich *p* = 0.001, medium vs. rich *p* < 0.001; eggs medium vs. rich *p* = 0.001; dairy medium vs. rich *p* = 0.001; MDD medium vs. rich *p* < 0.001; All-5 poor vs. rich *p* = 0.003, medium vs. rich *p* < 0.001. ^5^ Benue rural vs. urban: dark green leafy vegetables *p* < 0.001; flesh foods *p* = 0.001; pulses, nuts, seeds *p* = 0.029. Benue wealth status: starchy staples poor vs. rich *p* = 0.003; dark green leafy vegetables medium vs. rich *p* = 0.007; flesh foods poor vs. rich *p* < 0.001, medium vs. rich *p* = 0.006; eggs poor vs. rich *p* = 0.008, medium vs. rich *p* = 0.041; dairy poor vs. rich *p* < 0.001, medium vs. rich *p* < 0.001. ^6^ Nasarawa rural vs. urban: starchy staples *p* = 0.018; dark green leafy vegetables *p* < 0.001; other vitamin A-rich fruits and vegetables *p* = 0.001; flesh foods *p* < 0.001; eggs *p* < 0.001; dairy *p* <0.001; MDD *p* < 0.001; All-5 *p* < 0.001. Nasarawa wealth status: starchy staples poor vs. rich *p* < 0.001; dark green leafy vegetables poor vs. rich *p* = 0.002, medium vs. rich *p* = 0.001; other vitamin A-rich fruits and vegetables poor vs. rich *p* < 0.001, medium vs. rich *p* < 0.001; flesh foods poor vs. rich *p* < 0.001, medium vs. rich *p* < 0.001; eggs poor vs. rich *p* < 0.001, medium vs. rich *p* < 0.001, poor vs. medium *p* < 0.001; dairy poor vs. rich *p* < 0.001, medium vs. rich *p* < 0.001; pulses, nuts, seeds poor vs. rich *p* = 0.005; MDD poor vs. rich *p* < 0.001, poor vs. medium *p* = 0.005, medium vs. rich *p* < 0.001; All-5 poor vs. rich *p* < 0.001, medium vs. rich *p* < 0.001. ^7^ Senegal rural vs. urban: dark green leafy vegetables *p* < 0.001; other vitamin A-rich fruits and vegetables *p* < 0.001; flesh foods *p* < 0.001; eggs *p* < 0.001; dairy *p* < 0.001; MDD *p* < 0.001; All-5 *p* < 0.001. Senegal wealth status: dark green leafy vegetables poor vs. medium *p* < 0.001, poor vs. rich *p* < 0.001, medium vs. rich *p* < 0.001; other vitamin A-rich fruits and vegetables poor vs. medium *p* < 0.001, poor vs. rich *p* < 0.001, medium vs. rich *p* < 0.001; flesh foods poor vs. medium *p* < 0.001, poor vs. rich *p* < 0.001, medium vs. rich *p* < 0.001; eggs poor vs. medium *p* < 0.001, poor vs. rich *p* < 0.001, medium vs. rich *p* < 0.001; dairy poor vs. medium *p* = 0.006, poor vs. rich *p* < 0.001; pulses, nuts, seeds poor vs. rich *p* = 0.001; MDD poor vs. medium *p* < 0.001, poor vs. rich *p* < 0.001, medium vs. rich *p* < 0.001; All-5 poor vs. medium *p* < 0.001, poor vs. rich *p* < 0.001, medium vs. rich *p* < 0.001. ^8^ Tanzania rural vs. urban: dark green leafy vegetables *p* < 0.001; other vitamin A-rich fruits and vegetables *p* < 0.001; flesh foods *p* = 0.016; eggs *p* < 0.001; dairy *p* < 0.001; pulses, nuts, seeds *p* < 0.001; MDD *p* < 0.001; All-5 *p* < 0.001. Tanzania wealth status: dark green leafy vegetables poor vs. rich *p* < 0.001, medium vs. rich *p* < 0.001; other vitamin A-rich fruits and vegetables poor vs. rich *p* < 0.001, poor vs. medium *p* < 0.001, medium vs. rich *p* < 0.001; flesh foods poor vs. medium *p* < 0.001, poor vs. rich *p* < 0.001; eggs poor vs. rich *p* < 0.001, medium vs. rich *p* < 0.001; dairy poor vs. rich *p* < 0.001, medium vs. rich *p* < 0.001; pulses, nuts, seeds poor vs. rich *p* < 0.001, medium vs. rich *p* = 0.016; MDD poor vs. rich *p* < 0.001, poor vs. medium *p* < 0.001, medium vs. rich *p* < 0.001; All-5 poor vs. medium *p* = 0.017, poor vs. rich *p* < 0.001, medium vs. rich *p* < 0.001.

**Table 4:** Multivariable associations with Minimum Dietary Diversity for women of reproductive age.

	Cameroon	Kenya	Adamawa	Benue	Nasarawa	Senegal	Tanzania
Area							
Urban	1.00	1.00	1.00	1.00	1.00	1.00	1.00
Rural	0.85 (0.67, 1.08)	0.65 (0.44, 0.95) ^†^	1.25 (0.81, 1.92)	1.50 (1.02, 2.20) ^†^	0.83 (0.56, 1.23)	0.89 (0.77, 1.04)	0.62 (0.48, 0.80) ^†^
Women’s age							
15–24 years	1.00	1.00	1.00	1.00	1.00	1.00	1.00
25–39 years	1.09 (0.84, 1.41)	0.97 (0.67, 1.40)	1.06 (0.81, 1.39)	0.81 (0.59, 1.11)	0.94 (0.68, 1.32)	1.03 (0.91, 1.15)	1.08 (0.83, 1.41)
≥40 years	1.24 (0.78, 1.98)	0.84 (0.49, 1.44)	1.18 (0.70, 1.99)	0.94 (0.49, 1.83)	0.67 (0.36, 1.26)	0.88 (0.70, 1.12)	1.48 (1.00, 2.20)
Women’s education							
None	1.00	1.00	1.00	1.00	1.00	1.00	1.00
Primary	1.12 (0.72, 1.76)	5.34 (1.76, 16.21) ^†^	0.74 (0.56, 0.99) ^†^	1.86 (1.28, 2.71) ^†^	0.91 (0.60, 1.38)	1.08 (0.92, 1.25)	1.41 (0.98, 2.01)
Secondary/higher	1.29 (0.86, 1.95)	10.40 (3.41, 31.75) ^†^	1.03 (0.78, 1.36)	1.49 (1.02, 2.17) ^†^	0.89 (0.58, 1.36)	1.44 (1.20, 1.74) ^†^	3.06 (1.98, 4.71) ^†^
Household wealth							
Poor	1.00	1.00	1.00	1.00	1.00	1.00	1.00
Medium	1.04 (0.76, 1.41)	1.55 (1.04, 2.31) ^†^	0.59 (0.31, 1.13)	1.00 (0.70, 1.42)	1.66 (1.17, 2.35) ^†^	2.81 (2.42, 3.25) ^†^	1.75 (1.34, 2.30) ^†^
Rich	1.04 (0.79, 1.36)	1.83 (1.21, 2.75) ^†^	1.01 (0.52, 1.95)	1.40 (1.03, 1.90) ^†^	3.58 (2.33, 5.52) ^†^	3.80 (3.34, 4.32) ^†^	4.59 (3.38, 6.22) ^†^

Reference categories: urban, 15–24 years, no education, poor. ^†^ *p* < 0.05.

**Table 5:** Noncommunicable disease-associated food consumption for women of reproductive age.

	Cameroon N = 2073	Côte d’Ivoire N = 242	Kenya N = 864	Adamawa N = 1283	Benue N = 1047	Nasarawa N = 1151	Tanzania N = 2692
	*n* (%)	*n* (%)	*n* (%)	*n* (%)	*n* (%)	*n* (%)	*n* (%)
Soft drinks	437 (21.1)	43 (17.8)	28 (3.2)	184 (14.3)	243 (23.2)	367 (31.9)	245 (9.1)
Baked/grain-based sweets	693 (33.4)	85 (35.1)	127 (14.7)	168 (13.1)	141 (13.5)	247 (21.5)	195 (7.2)
Other sweets	727 (35.1)	170 (70.2)	668 (77.3)	622 (48.5)	311 (29.7)	674 (58.6)	1234 (45.8)
Processed meat	42 (2.0)	6 (2.5)	4 (0.5)	47 (3.7)	18 (1.7)	49 (4.3)	8 (0.3)
Unprocessed red meat	325 (15.7)	43 (17.8)	155 (17.9)	534 (41.6)	390 (37.2)	603 (52.4)	416 (15.5)
Deep-fried food	248 (12.0)	69 (28.5)	200 (23.1)	166 (12.9)	155 (14.8)	287 (24.9)	860 (31.9)
Fast food/instant noodles	121 (5.8)	5 (2.1)	7 (0.8)	223 (17.4)	139 (13.3)	246 (21.5)	28 (1.0)
Packaged ultra-processed salty snacks	44 (2.1)	4 (1.7)	4 (0.5)	52 (4.1)	37 (3.5)	93 (8.1)	39 (1.4)

**Table 6:** NCD-Protect, NCD-Risk, and GDR scores for women of reproductive age.

	Cameroon	CIV	Kenya	Adamawa	Benue	Nasarawa	Tanzania
NCD-Protect ^†^	2.76 (2.69, 2.83)	4.23 (4.01, 4.44)	3.18 (3.08, 3.29)	3.81 (3.68, 3.93)	3.60 (3.47, 3.73)	4.34 (4.20, 4.47)	3.14 (3.07, 3.20)
NCD-Risk ^‡^	1.29 (1.23, 1.35)	1.78 (1.59, 1.97)	1.38 (1.32, 1.45)	1.60 (1.50, 1.69)	1.39 (1.28, 1.49)	2.28 (2.16, 2.40)	1.13 (1.08, 1.17)
GDR ^¥^	10.47 (10.40, 10.54)	11.45 (11.25, 11.64)	10.80 (10.70, 10.91)	11.21 (11.10, 11.31)	11.21 (11.09, 11.32)	11.07 (10.95, 11.18)	11.01 (10.95, 11.07)

NCD, noncommunicable disease; GDR, Global Dietary Recommendations. ^†^ Denotes average consumption across nine health-protective food groups (whole grains; pulses; nuts and seeds; vitamin A-rich orange vegetables; dark green leafy vegetables; other vegetables; vitamin A-rich fruits; citrus; and other fruits); range 0–9. ^‡^ Denotes average consumption across eight food groups to limit or avoid (soft drinks; baked/grain-based sweets; other sweets; processed meat; unprocessed red meat; deep-fried food; fast food and instant noodles; packaged ultra-processed salty snacks); range 0–9 with processed meat double-weighted. ^¥^ Calculated as (NCD-Protect − NCD-Risk) + 9; range 0–18.

## Data Availability

The data presented in this study are available on request from the corresponding author. The data are not publicly available due to privacy or ethical restrictions.
